# Analysis of Risk Factors on Readmission Cases of COVID-19 in the Republic of Korea: Using Nationwide Health Claims Data

**DOI:** 10.3390/ijerph17165844

**Published:** 2020-08-12

**Authors:** Woo-Hwi Jeon, Jeong Yeon Seon, So-Youn Park, In-Hwan Oh

**Affiliations:** 1 Department of Preventive Medicine, School of Medicine, Kyung Hee University, Seoul 02447, Korea; emmanuel.woong@gmail.com (W.-H.J.); jeongyeon210@naver.com (J.Y.S.); 2 Department of Medical Education and Humanities, School of Medicine, Kyung Hee University, Seoul 02447, Korea

**Keywords:** COVID-19, readmission, risk factors

## Abstract

In South Korea, 4.5% patients of severe acute respiratory syndrome coronavirus 2 (SARS-CoV-2) were readmitted to hospitals after discharge. However, there is insufficient research on risk factors for readmission and management of patients after discharge is poor. In this study, 7590 confirmed coronavirus disease (COVID-19) patients were defined as a target for analysis using nationwide medical claims data. The demographic characteristics, underlying diseases, and the use of medical resources were used to examine the association with readmission through the chi-square test and then logistic regression analysis was performed to analyze factors affecting readmission. Of the 7590 subjects analyzed, 328 patients were readmitted. The readmission rates of men, older age and patients with medical benefits showed a high risk of readmission. The Charlson Comorbidity Index score was also related to COVID-19 readmission. Concerning requiring medical attention, there was a higher risk of readmission for the patients with chest radiographs, computed tomography scans taken and lopinavir/ritonavir at the time of their first admission. Considering the risk factors presented in this study, classifying patients with a high risk of readmission and managing patients before and after discharge based on priority can make patient management and medical resource utilization more efficient. This study also indicates the importance of lifestyle management after discharge.

## 1. Introduction

The novel coronavirus disease (COVID-19), which originated in China in December 2019, had recorded 6,535,354 confirmed cases and 387,155 deaths globally as of 15 May, 2020 [1]. South Korea saw a decline in the number of confirmed cases from the peak of 595 new cases on March 1, but the number has been increasing recently, especially around the capital region [2]. Since COVID-19 currently has no vaccine or treatment and the severe acute respiratory syndrome coronavirus 2 (SARS-CoV-2), which causes the disease, shows different patterns from the previously known coronaviruses, researchers are trying to understand the detailed characteristics of the virus and the disease through epidemiological investigations and case studies of patients. It is important to understand the characteristics from various aspects to prepare for the uncertainty of the COVID-19 situation.

Recently, with the number of cases of readmission increasing abroad, studies analyzing the clinical characteristics and risk factors of those patients have been carried out. According to a study by Jie Chen et al. that analyzed the risk factors and clinical outcomes of readmitted COVID-19 patients among 1087 patients in Wuhan, China, 7.6% of the patients were readmitted after discharge [3]. In addition, Xian et al. found that 27 out of 285 hospitalized COVID-19 patients in Guangdong, China tested positive again after recovering [4]. In a study by Somani S et al. that investigated 2864 COVID-19 patients admitted to five hospitals in New York, 103 patients (3.6%) tested positive again after discharge and were readmitted to the hospitals [5].

According to a previous study on high-risk readmitted patients, intensive management and a period of self-isolation after discharge seem to be effective measures to prevent readmission [6]. However, there have been only a few studies on readmitted patients, so little is known about the clinical effects of re-hospitalization on the patients’ prognosis or risk factors related to readmission [7]. In addition, studies conducted so far have targeted specific regions or hospitals, with small sample sizes, so the lack of representativeness is one of the limitations.

Readmission due to COVID-19, as suggested in previous studies, may be due to various reasons, such as false negative reverse transcription polymerase chain reaction (RT-PCR), prolonged viral clearance, and discharge despite not satisfying the criteria [8]. However, as the causes of readmission have not yet been clearly identified and only assumptions exist, it is necessary to analyze the risk factors.

As of 15 May, 447 patients in South Korea tested positive again or were readmitted to hospitals, accounting for 4.5% of those that ended self-isolation [9]. In Korea, there is insufficient research on risk factors for readmission, and, compared with the preventive measures for COVID-19, management of patients after discharge is poor. This is especially true as management of hospital beds could be a problem if a large number of patients are admitted all at once, so there is a need to maintain fewer readmissions. Therefore, we aimed to analyze the risk factors of patients who were readmitted after discharge based on a data set that could represent the national population, i.e., data on medical expenses related to COVID-19. This study has implications for post-discharge management that considers the risk factors, and also the efficient allocation and use of medical resources, including hospital beds, in the context of the spread of uncertain infectious diseases.

## 2. Materials and Methods

The South Korean government provides anonymized COVID-19 patient data sets to researchers in both Korea and abroad through COVID-19 International Research. The data source of the COVID-19 patient data set is the medical bill claims data by the Health Insurance Review and Assessment Service, where the medical use history of all citizens is accumulated based on the fee-for-service model. The COVID-19 patient data set is the claims data filed with the Health Insurance Review and Assessment Service by 15 May, 2020 and includes medical bills of 234,427 patients related to COVID-19, including suspected patients. The Health Insurance Review and Assessment Service identified 7590 confirmed patients by connecting the claims with the list of confirmed COVID-19 patients from the Korea Centers for Disease Control and Prevention (KCDC), and this information was also provided in the COVID-19 patient data set.

The 7590 confirmed COVID-19 patients were defined as targets for analysis. Out of 7950 patients, 7157 patients were admitted and 433 patients received ambulatory care. Though COVID-19 patients should be admitted in Korea, in the early outbreak of coronavirus, some patients stayed at home, receiving ambulatory care after a RT-PCR test [10] because of the shortage of hospital beds [11]. Patients who were admitted due to COVID-19 could discharge when the clinical criteria and test result were met [12].

Definition of readmitted patients for our analysis is based on the admission medical records. We have considered those with in-patient admission records related to COVID-19 three days after the date of discharge marked in the initial admission record.

After reviewing the patients’ demographic and clinical characteristics, logistic regression analysis was performed to analyze factors affecting readmission.

**Figure 1:**
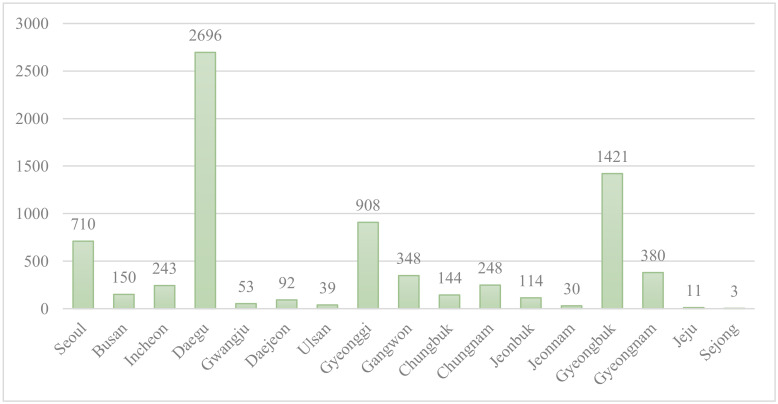
Coronavirus disease (COVID-19) patients by region in Korea.

The differences in demographic characteristics, underlying diseases, and the use of medical resources were described depending on whether the confirmed COVID-19 patients were readmitted or not, and the association with readmission was confirmed through the chi-square test. Gender, age, insurance eligibility, and the location of the medical institution were considered as demographic characteristics. Age was classified into groups of 0–19 years old, 20–39 years old, 40–64 years old, and 65 years old or older. For insurance eligibility, the patients were divided into health insurance subscribers and medical aid subscribers to indicate the patients’ economic status. In South Korea, health insurance subscribers pay their health insurance premiums in proportion to their income, while medical aid subscribers, due to their low-income status, do not pay for health insurance, so this was used as a variable that reflects economic status. The location of the medical institutions was divided into Daegu, Gyeongsangbuk-do, and other regions, and the difference in readmission rates between Daegu and Gyeongsangbuk-do, which was the source of a COVID-19 cluster infection, and other regions was compared (Figure 1). Underlying diseases included cancer, chronic obstructive pulmonary disease (COPD), ischemic heart disease, hypertension, diabetes, heart failure, chronic kidney disease, stroke, asthma, dementia, Parkinson’s disease, and liver cirrhosis, and the Charlson Comorbidity Index (CCI) score was reviewed. All underlying diseases and CCI scores were defined according to whether the patients had been diagnosed with the diseases more than once within one year from the time of the COVID-19 diagnosis. As variables for the use of medical resources, prescription of Kaletra, prescription of hydroxychloroquine, chest radiography, chest computed tomography (CT) scan, hospitalization in intensive care unit (ICU), and the length of stay, were considered. In Korea, using Kaletra and Hydroxychloroquine may be considered for all COVID-19 confirmed cases, but especially for groups who are at high risk [13].

Since this study sought to understand the risk factors of readmission, the variables related to the use of medical resources were from the first hospitalization, and the information from readmission was not used.

Logistic regression analysis was performed to analyze the risk factors affecting readmission due to COVID-19. The outcome variable was whether the patient was readmitted, and the explanatory variables were gender, age, insurance eligibility, location of medical institution, CCI score, prescription of Kaletra, prescription of hydroxychloroquine, chest X-rays, chest CT, ICU hospitalization, and the length of stay. SAS Enterprise Guides 4.3 (SAS Institute Inc., North Carolina, NC, USA) was used for all statistical analyses, and statistical significance was judged based on the value of 0.05.

This study was reviewed by the Kyung Hee University Institutional Review Board (IRB No. KHSIRB-20-164), and written consent was not required because the data used were anonymized public data provided by the Health Insurance Review and Assessment Service.

## 3. Results

### 3.1. Demographic and Clinical Characteristics of COVID-19 Readmission Patients

Of the 7590 subjects analyzed in South Korea, 328 patients were readmitted. In order to examine the characteristics of the patients that can be correlated with COVID-19 readmission, chi-square test on readmission was conducted, and the results are as follows (Table 1): Gender, age, insurance eligibility, and the location of the medical institution, which were considered as demographic characteristics, were all associated with the readmission of COVID-19 patients. The readmission rate of men was 5.5%, higher than that of women (3.5%). Older age was also associated with a higher readmission rate. The readmission rate of patients with medical benefits (15.4%) was about 4.7 times higher than that of patients with health insurance (3.3%). In terms of the location of medical institutions, the readmission rate in Gyeongsangbuk-do was the highest at 10.3%, while that of Daegu (2.9%) and other regions (3.0%) were similar.

Hypertension, diabetes, dementia, and Parkinson’s disease were associated with COVID-19 readmission, and the readmission rate of patients with those underlying diseases was high. There were 2461 patients with more than one underlying disease. The readmission rate of patients with underlying diseases was two times higher than those without an underlying disease. The CCI score, which indicates the severity of the underlying disease, was also related to COVID-19 readmission; the higher the CCI score, the higher the readmission rate.

For variables related to the use of medical resources, significant differences in the readmission rate were observed in all variables except hospitalization in the ICU. The readmission rate of patients who were prescribed Kaletra and hydroxychloroquine, which were prescribed for high-risk and severe patients, was 5.6% and 3.0%, respectively; the readmission rate of patients that were prescribed Kaletra had a higher readmission rate, whereas those who were prescribed hydroxychloroquine had a lower readmission rate than the patients who were not prescribed with the medicines. The patients that underwent chest radiographs or CT scans had a higher readmission rate than those who did not get the tests, with 5.2% of the patients that got a chest X-ray and 5.6% of those who underwent CT being readmitted. The median length of stay in first admission for all patients was 17.0 days (interquartile range: 10.0–24.0 days), the median length of stay in first admission for the readmitted patients was 9.0 days (interquartile range: 1.0–18.0 days), and the median length of stay in first admission for patients with no readmission was 17.0 days (interquartile range: 10.0–24.0 days), indicating that the length of stay in first admission for readmitted patients was shorter.

### 3.2. Results of Logistic Regression

The results of logistic regression analysis on the COVID-19 readmission patients are as follows (Table 2): In terms of demographic characteristics, being male (Odds Ratio (OR): 1.340, 95% CI: 1.055, 1.706), being 65 years of age or older (OR: 2.235, 95% CI: 1.111–4.497), having medical benefits (OR: 2.757, 95% CI: 2.040–3.725), and living in Gyeongsangbuk-do (OR: 2.876, 95% CI: 2.144–3.857) were associated with a higher risk of readmission due to COVID-19.

For underlying diseases, the CCI scores that describe the severity of the underlying diseases were reflected in the COVID-19 readmission model without considering the diseases separately. Logistic regression analysis confirmed that the higher the score for underlying diseases, the greater the risk of readmission due to COVID-19.

Patients that were prescribed Kaletra at the time of their first admission (OR: 1.388, 95% CI: 1.056–1.827) had a higher risk of readmission than those that were not prescribed the medicine. In addition, there was a higher risk of readmission for the patients with chest radiographs (OR: 1.591, 95% CI: 1.121–2.258) and CT taken (OR: 1.330, 95% CI: 1.031–1.717). The patients with a shorter length of stay in first admission had a higher risk of readmission (OR: 0.945, 95% CI: 0.933–0.958).

## 4. Discussion

This study used medical data to determine patients’ readmission status, compared the demographic characteristics, underlying diseases, and the use of medical resources based on whether COVID-19 patients were readmitted or not, and analyzed the risk factors that affect readmission. The analysis confirmed that 328 patients were readmitted, accounting for 4.3% of all subjects. In terms of demographic characteristics, males, those aged 65 years or older, medical aid subscribers, and those in Gyeongsangbuk-do had higher rates of readmission. Higher CCI scores for underlying diseases were also linked to a higher rate of readmission. In terms of the characteristics related to the use of medical resources, patients with Kaletra prescription, chest X-ray, and chest CT had a higher rate of readmission. A shorter length of stay at the time of first admission was associated with a higher rate of readmission.

The characteristics identified in this study are as follows: First, the risk of readmission was higher in high-risk elderly patients and males [14]. In this study, the risk of readmission was found to increase with age, and a statistically significant value (OR: 2.235, 95% CI: 1.111–4.497) was obtained in the group of people over 65 years of age. A survey of 72,314 COVID-19 patients in China found that the risk of readmission increased with age [15]. In addition, the finding is consistent with a report from New York City that revealed that 3.3% of 676 admitted patients aged 65 and over required re-hospitalization, confirming that the risk of readmission is higher in elderly patients than in other age groups [16]. In the elderly, the response of immune cells against the virus is reduced, so the virus can last longer in the body [17]. Therefore, there is a possibility that the patients were readmitted because the virus, which remained in the body [8], could have manifested again with symptoms or been redetected in RT-PCR [18].

By gender, men were 1.340 times more likely to be readmitted due to COVID-19 than women. Somani S et al. found that out of 103 readmitted patients, 65 were men, two times more than women [5], and a study by Lina Marcela Parra Ramírez et al. on COVID-19 readmitted patients showed that more men were readmitted due to COVID-19 [19]. Previous studies found differences in the effects of COVID-19 by gender. First of all, the immune response to the coronavirus is more active in women than in men, which allows the virus to remain in the men’s bodies for a long time [20,21]. It may suggest the possibility that the coronavirus, which remains in the body for a long time, was detected again during the RT-PCR re-testing. In addition, in male patients, the prevalence of chronic diseases, a coronavirus risk factor, is higher, and men have a higher rate of dangerous behaviors, such as tobacco and alcohol use, than females [22], which may have contributed to the negative effects of the coronavirus.

Furthermore, income level, which is an important factor for determining the level of health [23], also affected the coronavirus hospital readmission rate. A lower level of income led to a higher mortality rate from COVID-19, according to the COVID-19 mortality study in Sweden [24]. In this study, the probability of readmission was higher in a group receiving medical aid. Even though additional research on the incidence/death rate of COVID-19 according to socioeconomic status is necessary, we can suggest that the management of health after discharge or the usual lifestyle of patients with low income [25] could have affected COVID-19 readmission.

Logistic regression analysis on the location of medical institutions confirmed that the readmission rate of COVID-19 patients in Gyeongsangbuk-do was 2.876 times higher than that in other regions. In Gyeongsangbuk-do, 20% of cases were from infection at high-risk group facilities [26], and considering that the rate of infection at high-risk group facilities was 7.8% in Daegu [27] and 1.7% in Seoul [28], we can confirm that the rate of cluster infection in medical institutions, mental hospitals, and facilities for the disabled was higher than that in other regions. Therefore, it is possible that the high risk of readmission in high-risk patients, such as the elderly, the disabled, and those with underlying disease in those facilities, may have affected the high rate of readmission in Gyeongsangbuk-do. The KCDC also recently announced a case of group readmission in a nursing home in Gyeongsangbuk-do [9].

Analysis of the use of medical resources and CCI score during the first admission to hospital showed that a patient’s underlying disease affects readmission. A correlation between COVID-19 readmission and underlying diseases has already been demonstrated in other studies. A study in Switzerland that observed the characteristics of two readmitted patients reported that the patients had coronary artery disease, atrial fibrillation, and aortic stenosis as underlying diseases [29], and a recent study on patients readmitted with COVID-19 to five hospitals in New York City reported that the probability of readmission is higher in patients with hypertension and chronic obstructive pulmonary disease [5]. The finding that the rate of readmission is higher for patients with hypertension as the underlying disease was also confirmed in a study in Spain [19]. In addition, a study on readmitted patients in Wuhan, China reported that patients with pulmonary fibrosis as the underlying disease had a higher risk of readmission [3]. In this study, it was found that the probability of readmission was 4.391 times higher in patients with a CCI score of 1 and 5.086 times higher in those with a CCI score of 2 or more, and the group that received chest X-ray/chest CT during the first admission had a higher rate of readmission. For suspected COVID-19 patients with respiratory symptoms in South Korea, chest X-ray is taken for early detection of pneumonia and observation of the progress of pneumonia, and chest CT is taken for severe patients that cannot wait for RT-PCR results or need urgent surgery or procedure for other diseases [30]; chest X-ray/chest CT imaging suggests that there is a high risk of readmission in patients with respiratory or other underlying diseases.

Looking at the administration of antiviral drugs, a patient group that received lopinavir/ritonavir had a higher rate of readmission. The guidelines for lopinavir/ritonavir prescription from the Korean Society of Infectious Diseases recommend that they be administered to severe patients with pneumonia or to those who are currently at a high risk (older age, presence of underlying diseases) and are likely to develop severe symptoms [13]. Therefore, the high rate of readmission in the lopinavir/ritonavir group, as indicated above, suggests that the probability of readmission is higher in patients who are older and have underlying diseases. In addition, the high risk of readmission in the lopinavir/ritonavir treatment group, although not statistically significant, has been reported in other studies [17,31], so it is necessary to discuss whether lopinavir/ritonavir is effective in COVID-19 treatment [32].

Lastly, the length of stay at the time of first admission was shorter in the readmitted patients (OR: 0.945, 95% CI: 0.933–0.958). A recent study in Spain by Lina Marcela Parra Ramírez et al. reported that the duration of symptom onset and the length of stay was shorter in the readmitted patient group [19]. This may suggest improper patient care after discharge [5] or hasty discharge before complete recovery [18].

## 5. Conclusions

Clinically, the importance of readmission is controversial. According to a survey conducted by the KCDC, 44.7% of the readmitted patients were hospitalized after testing positive again in the RT-PCR test due to re-expression of COVID-19 symptoms (cough, sore throat, etc.). As the number of patients with recurrence of symptoms increased, the KCDC conducted a complete enumeration survey and found that 59.6% were those that were readmitted after being screened and testing positive again, regardless of symptoms. In May, the Korean government relaxed the standards for managing readmitted patients, as active monitoring of readmitted patients and contacts, epidemiological studies, and virological studies did not provide any evidence that readmitted patients have infectivity [9].

Therefore, this study aimed to identify the characteristics and risk factors of readmission, considering the impact on hospital bed usage rather than clinical effects. Therefore, it was found that 4.3% were readmitted due to COVID-19, with men, medical aid subscribers, the elderly, and those with underlying disease being more likely to be readmitted.

The limitations of this study are as follows: First, because the topic of readmission due to COVID-19 was operatively selected for this study, there exists a limitation in that the concept may have been reduced or expanded and cannot be standardized. In addition, although medical resources such as treatment options and drug prescriptions were known in this study, we could not provide more detailed information such as the clinical characteristics of the patients, because clinical results after the treatment were unknown. Another limitation is that COVID-19 infection is still prevalent, and this study was conducted in the middle of the pandemic, so there exists a possibility that the results might be different from the characteristics that may arise after the pandemic. Additional case studies as well as clinical studies of readmitted patients are necessary to supplement this study.

Nevertheless, this study is a case study of readmitted patients with a sufficient sample derived from national data, and the fact that it identifies the characteristics and risk factors of readmitted patients and suggests patient management standards makes it significant. Considering the risk factors presented in this study, especially for elderly patients and patients with underlying diseases, classifying patients with a high risk of readmission and managing patients before and after discharge based on priority can make patient management and medical resource utilization more efficient. In particular, the fact that there is a difference in the readmission rate depending on income level, in addition to clinical characteristics, indicates the importance of lifestyle management after discharge.

## Figures and Tables

**Table 1:** Demographic and clinical characteristics of coronavirus disease (COVID-19) readmission patients.

Patient Characteristics		Total (N)	Readmission		Non-Readmission		*p*-Value
			N	%	N	%	
Total		7590	328	4.3	7262	95.7	-
Sex	Male	3095	170	5.5	2925	94.5	<0.0001 **
	Female	4495	158	3.5	4337	96.5	
Age	0–19	431	10	2.3	421	97.7	<0.0001 **
	20–39	2629	70	2.7	2559	97.3	
	40–64	3165	151	4.8	3014	95.2	
	≥65	1365	97	7.1	1268	92.9	
Healthcare Coverage	Health insurance	6961	231	3.3	6730	96.7	<0.0001 **
	Medical aid	629	97	15.4	532	84.6	
Provider region	Daegu	2696	79	2.9	2617	97.1	<0.0001 **
	Gyeongbuk	1421	146	10.3	1275	89.7	
	Others	3473	103	3.0	3370	97.0	
Cancer	No	7308	312	4.3	6996	95.7	0.2551
	Yes	282	16	5.7	266	94.3	
COPD	No	7031	296	4.2	6735	95.8	0.0901
	Yes	559	32	5.7	527	94.3	
Ischemic heart disease	No	7440	321	4.3	7119	95.7	0.8337
	Yes	150	7	4.7	143	95.3	
Hypertension	No	6627	255	3.8	6372	96.2	<0.0001 **
	Yes	963	73	7.6	890	92.4	
Diabetes	No	6986	282	4.0	6704	96.0	<0.0001 **
	Yes	604	46	7.6	558	92.4	
Heart failure ^†^	No	7543	325	4.3	7218	95.7	0.4565
	Yes	47	3	6.4	44	93.6	
Chronic kidney disease ^†^	No	7548	324	4.3	7224	95.7	0.1063
	Yes	42	4	9.5	38	90.5	
Stroke	No	7427	317	4.3	7110	95.7	0.1234
	Yes	163	11	6.7	152	93.3	
Asthma	No	7395	318	4.3	7077	95.7	0.5746
	Yes	195	10	5.1	185	94.9	
Dementia	No	7357	304	4.1	7053	95.9	<0.0001 **
	Yes	233	24	10.3	209	89.7	
Parkinson’s disease ^†^	No	7544	322	4.3	7222	95.7	0.0135 *
	Yes	46	6	13.0	40	87.0	
Liver cirrhosis	No	7238	317	4.4	6921	95.6	0.2583
	Yes	352	11	3.1	341	96.9	
Number of underlying diseases	0	4949	157	3.2	4792	96.8	<0.0001 **
	≥1	2641	171	6.5	2470	93.5	
CCI Score	0	7500	301	4.0	7199	96.0	<0.0001 **
	1	75	22	29.3	53	70.7	
	≥2	15	5	33.3	10	66.7	
Lopinavir/ritonavir use	No	4976	181	3.6	4795	96.4	<0.0001 **
	Yes	2614	147	5.6	2467	94.4	
HCQ use	No	5599	269	4.8	5330	95.2	0.0005
	Yes	1991	59	3.0	1932	97.0	
Chest X-ray	No	2172	45	2.1	2127	97.9	<0.0001 **
	Yes	5418	283	5.2	5135	94.8	
Chest CT	No	5219	177	3.4	5042	96.6	<0.0001 **
	Yes	2699	151	5.6	2548	94.4	
ICU admission	No	6794	290	4.3	6504	95.7	0.5070
	Yes	796	38	4.8	758	95.2	
Length of stay in first admission, days ^‡^		17.0 (10.0–24.0)	9.0 (1.0–18.0)		17.0 (10.0–24.0)		<0.0001 **

Abbreviations: COPD, chronic obstructive pulmonary disease; CCI Score, Charlson Comorbidity Index Score; HCQ, hydroxychloroquine; CT, computed tomography; ICU, intensive care unit. * *p*-value < 0.05, ** *p*-value < 0.01, ^†^ Fisher’s exact test, ^‡^ Wilcoxon test, Length of stay in first admission is the median value (interquartile range).

**Table 2:** Logistic regression results for readmission of COVID-19 patients.

Patient Characteristics		Crude OR (95% CI)	Adjusted OR (95% CI) **
Sex	Female	1	1
	Male	1.595 (1.279, 1.992) *	1.340 (1.055, 1.706) *
Age	0–19	1	1
	20–39	1.152 (0.589, 2.252)	1.146 (0.581, 2.260)
	40–64	2.109 (1.103, 4.032) *	1.565 (0.802, 3.053)
	≥65	3.22 (1.664, 6.232) *	2.235 (1.111, 4.497) *
Healthcare Coverage	Health insurance	1.000	1.000
	Medical aid	5.312 (4.125, 6.842) *	2.757 (2.040, 3.725) *
Provider region	Others	1	1
	Daegu	0.988 (0.733, 1.330)	1.017 (0.736, 1.405)
	Gyeongbuk	3.747 (2.888, 4.861) *	2.876 (2.144, 3.857) *
CCI Score	0	1	1
	1	9.931 (5.962, 16.543) *	4.391 (2.254, 8.552) *
	≥2	11.961 (4.063, 35.209) *	5.086 (1.532, 16.889) *
Lopinavir/ritonavir use	No	1	1
	Yes	1.579 (1.263, 1.973) *	1.388 (1.056, 1.827) *
Chest X-ray	No	1	1
	Yes	49.557 (12.334, 199.112) *	1.591 (1.121, 2.258) *
Chest CT	No	1	1
	Yes	1.731 (1.386, 2.163) *	1.330 (1.031, 1.717) *
ICU admission	No	1	1
	Yes	1.124 (0.795, 1.590)	1.017 (0.692, 1.495)
Length of stay in first admission		0.922 (0.909, 0.935) *	0.945 (0.933, 0.958) *

Abbreviations: COPD, chronic obstructive pulmonary disease; OR, Odds Ratio; CI, Confidence Interval; CCI Score, Charlson Comorbidity Index Score; HCQ, hydroxychloroquine; CT, computed tomography; ICU, intensive care unit. * *p*-value < 0.05, ** Adjusted for hydroxychloroquine use.
